# Synthesis of Silica Microspheres—Inspired by the Formation of Ice Crystals—With High Homogeneous Particle Sizes and Their Applications in Photonic Crystals

**DOI:** 10.3390/ma11102017

**Published:** 2018-10-18

**Authors:** Xiaoyi Chen, Hongbo Xu, Chunxia Hua, Jiupeng Zhao, Yao Li, Ying Song

**Affiliations:** 1 School of Chemistry and Chemical Engineering, Harbin Institute of Technology, Harbin 150001, China; cxy84828@126.com (X.C.); iamxhb@hit.edu.cn (H.X.); luwanxibei@163.com (C.H.); songy@hit.edu.cn (Y.S.); 2 Center for Composite Materials and Structure, Harbin Institute of Technology, Harbin 150001, China

**Keywords:** silica microspheres, homogeneous nucleation, self-assembly, photonic crystals

## Abstract

Silica microspheres (SMs) must possess the performances of desirable monodispersity, narrow particle size distribution, and high sphericity for preparing photonic crystals (PCs) and other materials such as microspheres reference material, etc. We have adopted the techniques of increasing reactant concentration and raising the temperature to improve the synthesis rate of SMs, gaining inspiration from the formation mechanism of ice crystals. SMs with uniform particle sizes (polydispersity index less than 0.05) and good spherical features were fabricated through homogeneous nucleation. The mathematical relationship between particle sizes of SMs and reactant concentrations is further fitted. High accuracy of the regression equation is verified by an F-test and verification experiment. Highly ordered PCs (the stacking fault is about 1.5%, and the point defect is about 10^−3^) with dense stacked opal structures have been obtained by self-assembly of SMs. In addition, highly ordered PCs (the stacking fault is about 3%, and the point defect is about 10^−3^) with non-dense packed opal structure and inverse opal structure were successfully prepared. PCs of inverse opal structure were used to examine their response characteristics to identify ethanol, exhibiting good performance. Our research may provide significant inspiration for the development of other sorts of microspheres.

## 1. Introduction

Three-dimensional photonic crystals (PCs) have unique and complicated structures, which enable the manipulation of the flow of light [1,2]. Furthermore, the special dispersion properties of PCs could produce diverse fantastic, interesting, and anomalous refraction effects [3,4,5], including negative refraction [6,7,8], self-collimation [9,10], and a superprism effect [11,12]. Consequently, PCs have many important applications in various fields [13,14]. In recent years, self-assembly technology of colloidal crystals has been an effective way to fabricate three-dimensional PCs, the band gap of which may range from the near-infrared to visible wave band. Self-assembly of colloidal crystals is considered to be the most promising method for the fabrication of three-dimensional PCs [15]. Self-assembly refers to the spontaneous process of forming a thermodynamically stable, structurally defined, and specific congeries between structural units such as molecules and nanoparticles through non-covalent interactions under equilibrium conditions. Monodisperse microspheres can be self-assembled in different ways to obtain different kinds, properties, and applications of PCs and other structures such as hollow sphere colloidal PCs [16], dendritic fibrous nano-silica PCs [17], large-area silica microspheres/nanospheres [18], and large-area flexible colloidal photonic crystal film [19,20]. Poly(styrene-methyl methacrylate-acrylic acid) (P(St-MMA-AA)) nanospheres were used to produced polymer colloidal crystals by self-assembly of refrigerated centrifugation [21]. Monodisperse polystyrene (PS) microspheres were used to fabricate colloidal crystals by vertical deposition [22]. Silica microspheres (SMs), because of their good chemical and thermal stability, are used as the optimal material for self-assembly of colloidal crystals [23,24,25]. SMs, as structure units of closed-packed arrangements of colloidal crystals, must possess the performances of desirable monodispersity, narrow particle size distribution, and high sphericity. It is difficult to assemble opal structure and further develop inverse opal structure PCs without producing high-quality SMs [26]. Since 1968, Stöber successfully prepared SMs with particle sizes of 50 to 2000 nm after systematically discussing the influence of the concentration of each component (esters system, alcohols system, water system, and alkali system) on synthesis velocity and particle size distribution [27]. Consequently, researchers have explored the mechanisms of nucleation and growth of SMs, and improved the Stöber method. Matsoukas and Gulariby determined the scattering intensity through dynamic light scattering to investigate the size variation regularity of SMs. They used a Raman spectrum to detect the oxethyl bond (C_2_H_5_O–), and discovered that hydrolysis occurs as a function of time. Furthermore, a monomer addition model was established to expound the mechanisms of nucleation and growth of SMs [28,29]. Zukoski and his collaborators established a sub-particle aggregation model by observing the morphology of SMs using a transmission electron microscope, and illustrated the mechanism of nucleation and growth [30,31]. Chen and others suggested that two kinds of fabrication and growth models (dehydroxylation and dealcoholysis) may all exist under the condition of controlling the growth of SMs using a seeding method, which is favorable to obtain a large particle size and monodisperse SMs [32,33]. Kim et al. prepared SMs with homogeneous particle sizes (as small as 23 nm) through continuous and semi-continuous addition of tetraethoxysilane (TEOS) [34]. Temperature and other factors affect the monodispersity, sphericity, particle sizes, and deviation of synthesized SMs by the Stöber method [35,36]. The existing synthesis techniques of SMs are still difficult to satisfy the demanding requirements for the fabrication of PCs that have a high roundness, better monodispersity, narrow particle size distribution, and adjustable particle sizes. Moreover, the particle size change in the synthesis process of SMs is highly random and could not be precisely controlled. 

We gain inspiration from the mechanism of the formation of ice crystals in nature in so far as the increase of the supercooling degree of water leads to a rising degree of deviation from the equilibrium state, which could greatly improve the probability of homogeneous nucleation. When temperature rapidly dropped to below the freezing point of water (higher subcooling degree), fine and uniform ice crystals are acquired (homogeneous nucleation). However, when the temperature is slowly reduced to below the freezing point of water (lower subcooling degree), heterogeneous nucleation occurs and uneven crystals can potentially develop [37]. 

In the process of synthesizing SMs by the Stöber method, Si(OH)_4_ solution at a critical nucleation concentration generated large quantities of crystal nucleus explosively, leading to the solution strength rapidly being diminished to below a critical nucleation concentration. As a result, new nuclei are no longer produced, crystal nuclei larger than the critical grain size stably exist and grow slowly to develop monodisperse SMs [38,39]. A competitive relationship between homogeneous nucleation and heterogeneous nucleation exists in fabricating monodisperse SMs [40,41,42,43]. When homogeneous nucleation occurs, it is easy to form SMs with uniform particle sizes, which is similar to the forming process of ice crystals. Inspired by uniform ice crystals which are developed during the rapid cooling process (higher supercooling degree), we used an improved Stöber method to synthesize SMs with a narrow size distribution, good spherical morphology, and excellent monodispersion, satisfying the strict requirements of fabricating PCs. The mathematical relation equation of the particle sizes with the ammonia, TEOS, and water content is established. Highly ordered PCs with dense stacked opal structure and non-dense packed opal structure have been successfully prepared. Then, on the basis of these, highly ordered PCs with non-dense stacked inverse opal structures were obtained, which exhibit good performance of response identification characteristics to ethanol. Monodisperse SMs, with desirable spherical morphology and controllable size, could be applied widely in the fields of micro-size standard materials, PCs, ordered macroporous materials, macroporous catalysts, and composite materials for proton exchange membranes.

## 2. Materials and Methods

### 2.1. Synthesis of SMs

TEOS, ethanol, ammonia, 3-aminopropyltriethoxysilane glutaraldehyde, polyethylene glycol diacrylate (PEGDA), and 2-hydroxy-2-methylpropiophenone were purchased from Sigma-Aldrich (Shanghai, China). The phenomenon of supercooling occurs when water remains in the liquid phase below the freezing point without freezing. In the process of water freezing, the subcooling degree has an important effect on the shape and size of ice crystals. Under the conditions of a high subcooling degree and rapid cooling, fine and uniform ice crystals are formed because of the augmented probability of homogeneous nucleation. SMs were synthesized using an improved Stöber method to promote the hydrolysis of TEOS and increase the probability of homogeneous nucleation. According to the concentration ratios, shown in Table S1, we synthesized SMs with different diameters. A certain amount of TEOS and 170 mL anhydrous ethanol were poured into a beaker and stirred for 5 min to obtain evenly mixed A solution. Then, a certain amount of ammonia and deionized water were poured into another beaker and stirred for 5 min to obtain evenly mixed B solution. B solution is added into the A solution and stirred for 24 h at a constant temperature (20 °C). The humidity ranged from 20% to 22%. Then, the mixture was repeatedly centrifuged and washed with anhydrous ethanol, and SMs powders were obtained after constant temperature drying.

### 2.2. Surface Modification of SMs

#### 2.2.1. Aminated Modification of SMs

SMs (10 mg), with particle size of 288 nm, were added to 50 mL of anhydrous ethanol, and were dispersed evenly using ultrasonication (Kunshan ultrasonic instruments, Kunshan, China). Then 35 μL of 3-aminopropyltriethoxysilane (APTES) was added into the above suspension and stirred at 25 °C for 24 h.

#### 2.2.2. Chemical Modification of SMs by the Carbonylation Method

The surface-aminated SMs (10 mg) were added to a certain amount of water as the dispersing agent, and were dispersed evenly using ultrasonication. Then 35 uL of glutaraldehyde was added into the above suspension and stirred at 25 °C for 12 h.

### 2.3. Preparation of Close-Packed PCs

SMs were dispersed into anhydrous ethanol to form a suspension, with a concentration of 40%, which was dropped on the hydrophilic glass uniformly under the condition of constant temperature (20 °C). The humidity ranged from 20% to 22%. With the evaporation of anhydrous ethanol, SMs formed close-packed PCs under the actions of capillary forces and surface tension.

### 2.4. Preparation of Non-Dense Packed PCs

SMs of 40 μL, using 2 mL ethanol as the dispersant, were dispersed evenly using ultrasonication. Polyethylene glycol diacrylate (PEGDA, 60 μL) and 0.6 μL of 2-hydroxy-2-methylpropiophenone were mixed with the above suspension and heated for 2 h at 90 °C. Then 30 μL of the mixture was removed and sandwiched between two glass slides (1.5 cm × 1.5 cm), and left to sit for 20 min. The mixture was solidified for 1 min using ultraviolet (UV) light, resulting in the formation of non-dense packed opal structure PCs. The photonic crystal film was removed from the glass slides after UV curing for 1 min, and immersed in the HF acid solution for 4 h (the SMs in the film were removed). Then the inverse opal photonic crystal films (PEGDA films) were dried after being washed repeatedly with anhydrous ethanol and deionized water. 

### 2.5. Identification of Ethanol Using the Inverse Opal Photonic Crystal Films

Reflection spectra (Maya2000 pro, Ocean Optical, Largo, FL, USA) of the inverse opal photonic crystal films were determined. After being submerged in the ethanol for 10 min, the reflectance spectra of the inverse opal photonic crystal films were measured once again. Ethanol was identified by comparing the change of reflectance spectra of the inverse opal photonic crystal films. 

### 2.6. Characterization of SMs and PCs

The particle sizes and morphology of SMs were determined by Scanning Electron Microscope (SEM, Supra55, ZEISS, Jena, Germany). The particle sizes of the microspheres were calculated using Image J Software ((V1.8.0, Bethesda, MD, USA) which is especially suitable for spherical nanoparticles. We took 30 intact microspheres from each SEM image, whose particles were measured and analyzed statistically. Particle sizes and polydispersity index (PDI) were determined through dynamic light scattering (Zetasizer Nano ZS90, Malvern Panalytical, Malvern, UK), the surface characteristics of SMs were characterized by Zeta potential (Zetasizer Nano ZS90). Aminylation and carbonylation were characterized by changing values of Zeta potential. The reflection spectra characteristics of PCs were determined by Ocean Optical Maya2000 pro Spectrometer. The formula for calculating the coefficient of variation of SMs is

(1) $$D=\frac{\delta }{d},\;\delta ={\left[\sum \frac{{\left(d_{i}-\bar{d}\right)}^{2}}{\left(n-1\right)}\right]}^{\frac{1}{2}}\;$$

where δ is the standard deviation, $d_{i}$ the diameter of a single particle, $\bar{d}$ the average diameter of particles, and n is the number of microspheres.

## 3. Results and Discussion

### 3.1. Synthesis and Characterization of Monodisperse SMs with Uniform Particle Sizes

**Figure 1:**
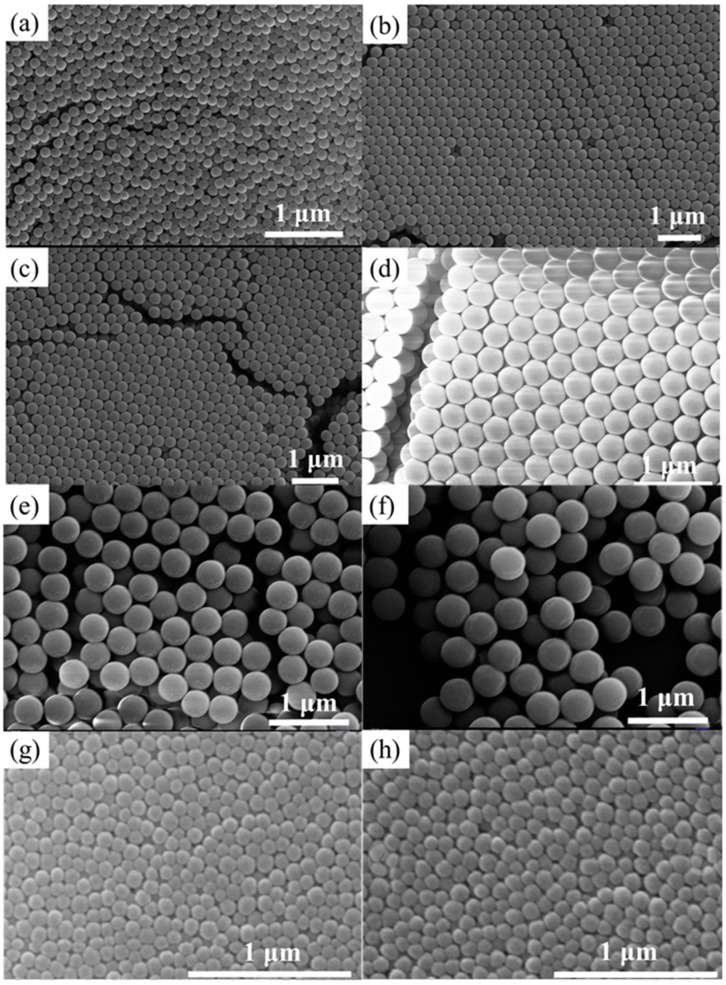
SEM images of (**a**) 186 nm silica microspheres (SMs); (**b**) 243 nm SMs; (**c**) 256 nm SMs; (**d**) 343 nm SMs; (**e**) 564 nm SMs; (**f**) 763 nm SMs; (**g**) 94 nm SMs; and (**h**) 86 nm SMs.

Figure 1 and Figure S1 show that SMs within the range of 105–763 nm present a good morphology and low coefficient of variation using an improved Stöber method. Average diameter statistic results, coefficient of variation (according to the Formula (1)), and PDI of synthetic SMs are shown in Table S2. From the above results, we can see that the SMs with particle sizes below 94 nm exhibit poor spherical morphologies, a larger coefficient of variation, and an increased PDI.

In the synthesis process, the formation of SMs with a uniform particle size and low coefficient of variation depends on the homogeneous nucleation, which only arises when the concentration of Si(OH)_4_ produced by TEOS hydrolysis and the polymerization rate of Si(OH)_4_ are high enough. We first analyzed the effect of reactant concentration on homogeneous nucleation. With the increase of reactant concentration (ammonia content), the hydrolysis of TEOS was promoted to produce Si(OH)_4_, the reaction rate increased, and the homogeneous nucleation was advanced. Meanwhile, the increment of ammonia content is conducive to the polymerization of Si(OH)_4_ to form the cross-linked structure of Si–O–Si chain, and also significantly promotes the aggregation and growth of the nucleus. Consequently, particle sizes of the generated SMs are all augmented with the increase of ammonia content.

According to the reaction equation, TEOS can be hydrolyzed completely when the molar ratio of TEOS to water is 1:4. TEOS could be incompletely hydrolyzed when the water content was lower; hence, the particle size augments with the increase of water content initially. However, when the water content increased further, the concentration of Si(OH)_4_ was diluted, and the reaction rate was reduced. As a result, the homogeneous nucleation was inhibited, resulting in a decrease of the hydrolysate Si(OH)_4_ concentration and the formation of a low degree of crosslinking Si–O–Si chain. Particle sizes of SMs reduced with the increase of water content.

A decreasing TEOS concentration may reduce the rate of TEOS hydrolysis and the polymerization rate of Si(OH)_4_, thus, the Si–O–Si chain with a low crosslinking degree is formed. Particle sizes of the SMs increased with the increase of TEOS content.

**Figure 2:**
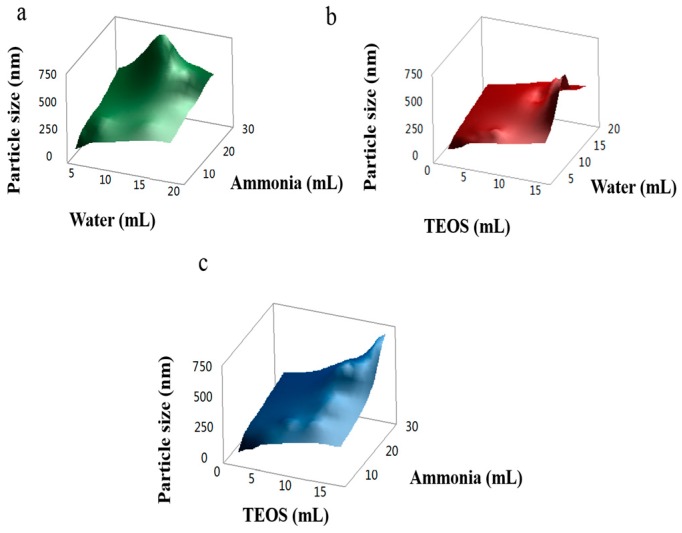
Effects of interactions between ammonia, tetraethoxysilane (TEOS), and water on the particle sizes of SMs: (**a**) Effect of interaction between water and ammonia on the particle sizes of SMs; (**b**) effect of interaction between TEOS and water on the particle sizes of SMs; (**c**) effect of interaction between TEOS and ammonia on the particle sizes of SMs.

We further analyzed the effect of interaction between ammonia, water, and TEOS on particle sizes and reaction rate. Figure 2 reveals that TEOS has the most significant effect on the growth of particle size and reaction rate, ammonia the second, and water has the least. SMs with homogeneous particle sizes could be obtained within the range of 105–763 nm by adjusting the amounts of ammonia, TEOS, and water. Meanwhile, homogeneous nucleation occurs, and the particle sizes of SMs are homogeneous (low coefficient of variation). However, heterogeneous nucleation of SMs with particle sizes below 94 nm arises, which results in a large coefficient of variation of particle sizes, see Table S2.

The quantitative relationship between the particle size of SMs and three factors (ammonia, TEOS, and water) is established as the formula:

(2) D=5.2+19.14x−6.54y+17.5z

where D is diameter of microspheres (nm), x is the content of TEOS (mL), y is the content of water (mL), and z is the content of ammonia (mL). Accuracy analysis results of fitting the regression equation were revealed in Table S3. 

**Figure 3:**
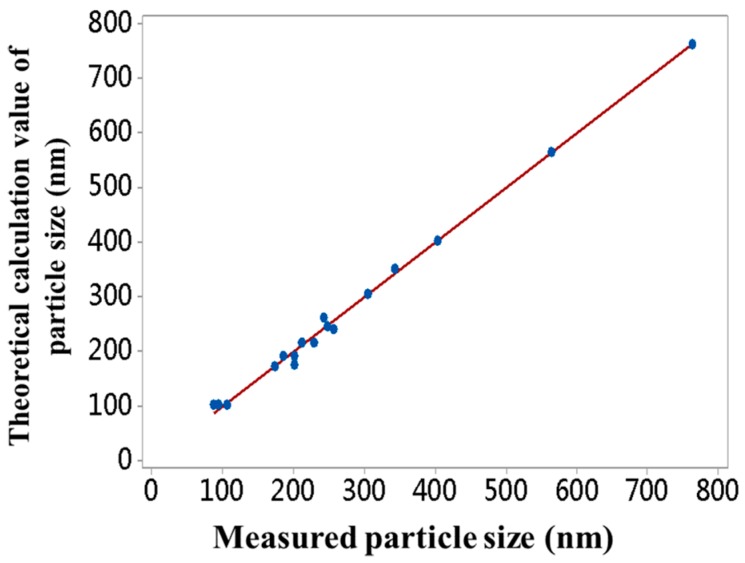
The relationship curve of the predicted values and the measured values of particle sizes.

The F-value of the regression equation is 336.75, which confirmed the high accuracy of the fitting equation. We further fitted the relationship between the actual particle sizes and the theoretical predicted particle sizes. Within the range of 86 nm to 763 nm, the regression equation between the actual and the theoretical predicted particle sizes is:

(3)  Y=1.183+0.9956X

where Y is the theoretically predicted value of particle size, X is the actual value of particle size (R^2^ = 0.996). There was a significant linear relationship between the two, see Figure 3. The theoretical prediction formula of particle sizes, SMs with arbitrary particle sizes in the range of 86 to 763 nm, can be obtained through adjusting the contents of ammonia, water, and TEOS, overcoming the randomness defects of particle sizes of SMs synthesized by the traditional Stöber method.

**Figure 4:**
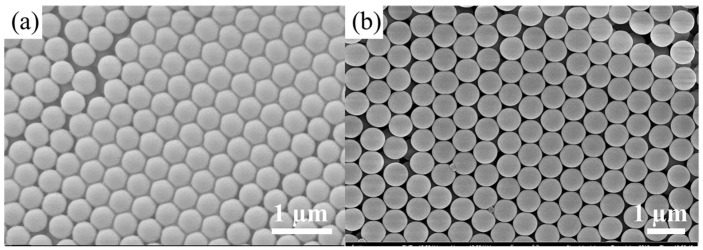
SEM images of synthesized SMs with particle sizes of (**a**) 451 nm and (**b**) 547 nm.

We randomly selected two concentration ratios, that is, ammonia 13 mL, TEOS 15 mL, and water 10 mL, and the theoretical and actual measured particle sizes were 456 nm (according to the Formula (2)) and 451 nm, respectively. The theoretical and actual measured particle sizes were 552 nm and 547 nm when the 15 mL of ammonia, TEOS 18 mL, and water 10 mL was used. Figure 4a,b shows that two groups of synthesized SMs were verified to have favorable spherical morphologies and monodispersity. The coefficient of variation of SMs is less than 7%. The high accuracy of the fitting regression equation was indicated in terms of the relationship between the particle sizes of the SMs and the concentrations of the reactants.

We can see the spherical morphologies of the SMs, as shown in Figure 1g,h, obtained by the concentration ratios of number 7 and number 4 of Table S1, which demonstrate that the coefficients of variation of particle sizes are higher. The primary reason for this was attributed to the reduction of the chemical reaction rate, due to lower amounts of ammonia and TEOS and the rise in water content. The decline in the concentration and polymerization rate of Si(OH)_4_ leads to heterogeneous nucleation. Meanwhile, in order to obtain a photonic bandgap in the ultraviolet region, the diameter of the SMs should be controlled to be below 100 nm. Improving the polymerization reaction rate of the Si(OH)_4_ by increasing the reaction temperature on the basis of controlling the contents of ammonia, TEOS, and water is necessary to acquire homogeneous SMs with smaller particle sizes. We selected the sample concentrations of number 7 and number 4 in Table S1, when the reaction rate was improved by increasing the reaction temperature to 60 °C, the acquired SMs present clearly decreased coefficients of variation (the PDI of 66 nm SMs is 0.048 and the PDI of 78 nm SMs is 0.043) and better spherical morphologies, see Figure S2, due to the increased rate of homogeneous nucleation. As the process of nucleation and polymerization is exothermic, the equilibrium moves to the negative direction with the rise in temperature, weakening the nucleation effect and particle growth of SMs. Therefore, the particle sizes of SMs acquired at 60 °C decreased compared with those at 20 °C (from 86 nm to 66 nm, 94 nm to 78 nm).

### 3.2. Surface Modification of SMs

SMs are adverse to dispersing evenly in an organic medium since hydroxyl is a typical hydrophilic group; therefore, it needs to be modified on the surface to solve the problems of dispersion and compatibility with an organic substrate. The zeta potential value of unmodified SMs was negative due to the weak interaction between the surface of SMs and water, which resulted in the formation of silicic acid. Further ionization of silicic acid lead to negative charges on the surface of SMs, see Figure S3a. The zeta potential value of SMs changed from a negative value to a positive value after amination, see Figure S3b, which indicated that the amino group had been successfully modified on the surface of SMs. On the basis of amination, the length of the surface carbon chain could be enlarged by the introduction of the aldehyde group. The negative zeta potential value substituted for the positive one, see Figure S3c, which demonstrated that the aldehyde group has been successfully cross-linked with the amino groups. The surface of SMs was negatively charged by the free aldehyde groups; as a result, the zeta potential value became negative.

**Figure 5:**
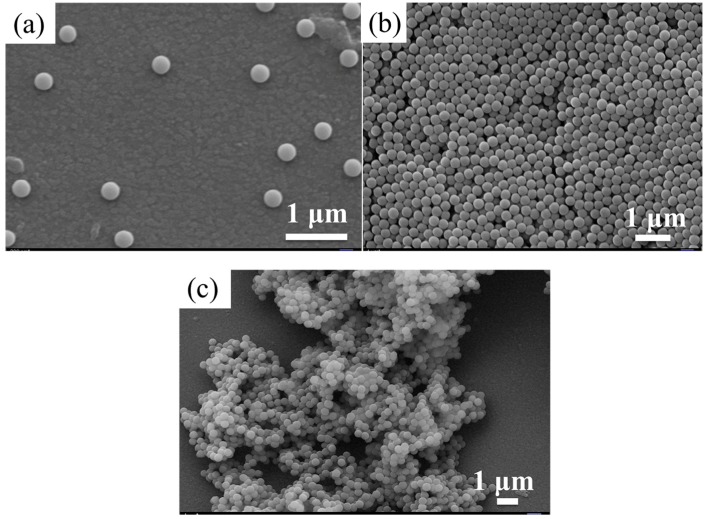
(**a**) The morphology of the original 288 nm SMs; (**b**) aminated modification of 288 nm SMs; (**c**) carbonylation modification of 288 nm SMs.

Figure 5a exhibits the excellent morphology and dispersibility of the original 288 nm SMs. After aminated modification, insignificant variation can be discovered in the microspheres’ morphology and dispersivity, see Figure 5b. In the process of surface amination, the amino group replaced the previous hydroxyl group, enlarging the length of the molecular chain. As the amino group carries an amount of positive electricity, the mutual exclusion of surface charges makes it difficult to agglomerate. After the introduction of aldehyde groups, the glutaraldehyde contains two carbonyl groups which are prone to form crosslinking network between the amino groups and SMs, resulting in the agglomeration of SMs, see Figure 5c. However, a suitable crosslinking degree would enhance the binding force and mechanical properties of SMs which could be further applied in organic-inorganic composite materials and other fields. 

### 3.3. Fabrication of Close-Packed Opal PCs

**Figure 6:**
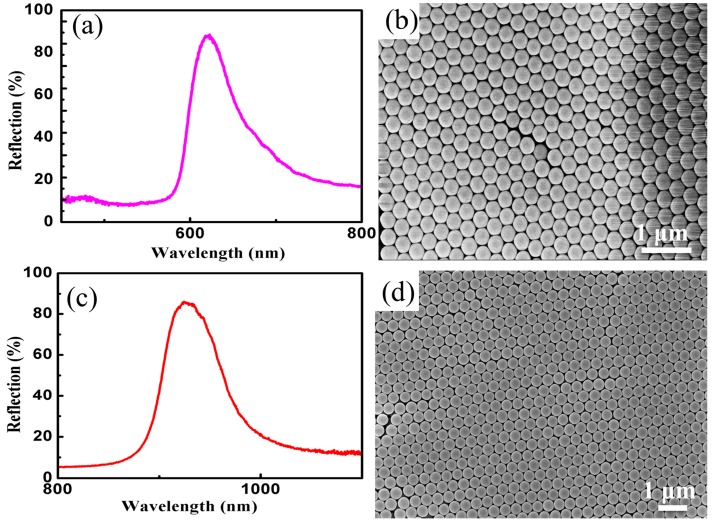
(**a**) Reflection spectrum of 283 nm SMs; (**b**) SEM image of photonic crystals (PCs) formed by self-assembly of 283 nm SMs; (**c**) Reflection spectrum of 427 nm SMs; (**d**) SEM image of PCs formed by self-assembly of 427 nm SMs.

Figure 6 reveals the reflection spectra of PCs formed by self-assembly of 283 nm and 427 nm SMs, and SEM images of PCs formed by self-assembly of 283 nm SMs and 427 nm SMs. The measured values of the reflective center wavelength are 635 nm and 958 nm, respectively, see Figure 6a,c. According to the Braggs-Snell formula, the theoretical calculation values of reflective center wavelength are 627 nm and 965 nm, respectively. The comparison between results calculated and those measured suggests that they are in fairly good agreement with each other. It can be seen from the SEM images, shown in Figure 6b,d, that PCs with a face-centered cubic (FCC) lattice are formed by SMs, presenting a higher ordering degree and fewer defects (the stacking fault is about 1.5% and the point defect is about 10^−3^). It is further demonstrated by the above results that synthetic SMs possess a low coefficient of variation of particle size and favorable spherical morphology.

### 3.4. Preparation of Non-Close-Packed Opal PCs and Inverse Opal PCs

**Figure 7:**
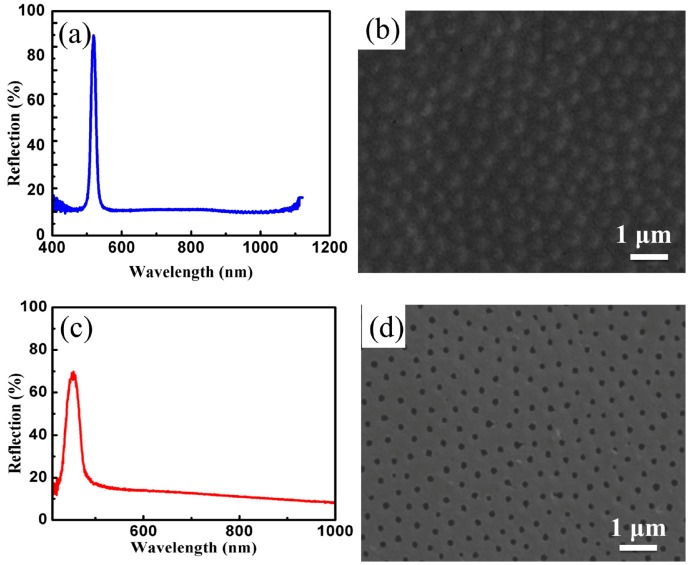
(**a**) Reflection spectrum of non-closed-packed opal structure PCs; (**b**) SEM image of non-closed-packed opal structure PCs; (**c**) Reflection spectrum of non-closed-packed inverse opal structure PCs; (**d**) SEM image of non-closed-packed inverse opal structure PCs.

The reflection spectrum and SEM image of opal PCs with a non-close-packed structure (the stacking fault is approximately 3%, and the point defect is about 10^−3^) are shown in Figure 7a,b. It can be seen from Figure 7a that the reflectivity of non-dense packed opal PCs is high, and the reflective bandwidth is narrow (the full width at half maximum is 8.2 nm), which mainly resulted from the similar refractive index between PEGDA and SMs (the refractive indexes of PEGDA and SMs are 1.47 and 1.46, respectively). From Figure 7b, we can see that the non-dense packed opal PCs possess the FCC structure. Figure 7c,d illustrates the reflection spectrum and SEM image of inverse opal PCs with a non-close-packed structure. It is clear that the three-dimensional ordered macroporous structure, see Figure 7d, of PEGDA is completely preserved after removing SMs, exhibiting the characteristics of reflected light, see Figure 7c. The reflection center wavelength of inverse opal PCs has a clear blueshift relative to the opal PCs, see Figure 7a,c, because the refractive index of the air is lower than that of the SMs. After removing SMs, the effective refractive index decreased and engendered the blueshift of the reflection spectrum, according to the Bragg-Snell formula. The bandwidth of reflected wavelength broadens because the difference of refractive index between air and PEGDA is large. 

### 3.5. Detection of Ethanol Using Inverse Opal Structure PCs

**Figure 8:**
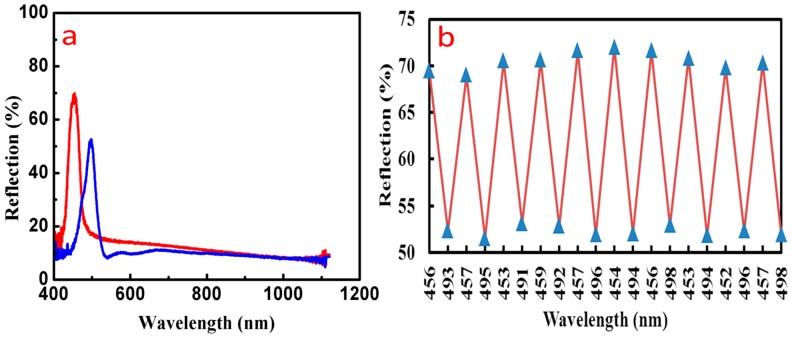
(**a**) Reflection spectrum of the inverse opal structure PCs before and after being filled with ethanol; (**b**) Spectral change of reflectivity and reflection wavelength after 10 ethanol-air cycles.

The voids of non-dense packed inverse opal PEGDA PCs are filled with air, which could be replaced by other liquids. Because the refractive index of the organic solvent is larger than that of the air, the effective refractive index of the PCs is increased, and the reflection center wavelength could present redshift when the non-dense stacked inverse opal PCs are immersed in organic solvents such as ethanol. Figure 8a indicates that the reflection center wavelength of the inverse opal structure has obvious redshift after being filled with ethanol. Thus, the effective refractive index of PCs is greatly improved and spectral redshift occurs, demonstrating good response characteristics and high resolution. Figure 8b shows the spectra of reflectivity and the reflection center wavelength had little change after 10 ethanol-air cycles, which indicates that the inverse opal PEGDA structure has favorable stability. Figure 8 suggests that the non-dense stacked inverse opal PCs have favorable response characteristics to ethanol. According to this principle, the non-dense stacked inverse opal PCs may also exhibit these response characteristics to other organic solvents such as styrene and tripropylene glycol. PEGDA inverse opal PCs are promising sensors for detecting other organic solvents in the field of environmental protection.

## 4. Conclusions

SMs with uniform particle sizes and good spherical shape were prepared, inspired by the formation mechanism of ice crystals in nature through homogeneous nucleation. In the range of 105 to 763 nm, raising the concentration of reactants could result in SMs with homogeneous particle sizes because of the accelerated rate of formation and polymerization of Si(OH)_4_. We analyzed the effect of the concentration of reactants on the particle size and reaction rate of SMs. We also obtained a low coefficient of variation of SMs with diameters below 94 nm through homogeneous nucleation resulting from raising the reaction temperature for the accelerated rate of formation and polymerization of Si(OH)_4_. In the range of 86 to 763 nm, the mathematical relationship between particle size of SMs and reactant concentration is further fitted. The regression equation with higher accuracy is verified by F-test and verification experiment, overcoming defects of the randomness of particle sizes in previous research. The acquired SMs with uniform particle sizes and good spherical features were used to fabricate highly ordered dense stacked opal PCs, non-dense stacked opal PCs, and non-dense stacked inverse opal PCs. Response identification characteristics to ethanol of non-dense packed inverse opal structure PCs were examined, exhibiting good performance. SMs could be applied widely in other fields such as micro-size standard materials.

## Supplementary Materials

The following are available online at http://www.mdpi.com/1996-1944/11/10/2017/s1, Figure S1: SEM images of silica microspheres, (a) 105 nm, (b) 174 nm, (c) 201 nm, (d) 201 nm, (e) 212 nm, (f) 228 nm, (g) 283 nm, (h) 305 nm, (i) 403 nm, Figure S2: SEM images of silica microspheres (a) 66 nm, (b) 78 nm, Figure S3: Zeta potential changes of silica microspheres before and after surface chemical modification. (a) Zeta potential of silica microspheres, (b) Zeta potential of aminated silica microspheres, (c) Zeta potential of silica microspheres after carbonylation, Table S1: Concentration ratios of ammonia, TEOS, and deionized water, Table S2: Statistical analysis of synthesized silica microspheres, Table S3: Accuracy analysis of fitting regression equation.
