# Supplementary material for: Synthesis of Silica Microspheres—Inspired by the Formation of Ice Crystals—With High Homogeneous Particle Sizes and Their Applications in Photonic Crystals
Source: Materials (Basel). 2018 Oct 18;11(10):2017. doi: 10.3390/ma11102017 (PMC6213217; doi:10.3390/ma11102017)
Supplement: Supplementary file 1 [file materials-11-02017-s001.docx]

Supplementary: Synthesis of Silica Microspheres—Inspired by the Formation of Ice Crystals—With high Homogeneous Particle Sizes and Their Applications in Photonic Crystals

Xiaoyi Chen, Hongbo Xu, Chunxia Hua, Jiupeng Zhao *, Yao Li * and Ying Song

**Table S1.** Concentration ratios of ammonia, TEOS, and deionized water.

| Numbers | Ammonia (mL) | Deionized water (mL) | TEOS(mL) |
| --- | --- | --- | --- |
| 1 | 10 | 6 | 5 |
| 2 | 10 | 7 | 5 |
| 3 | 7 | 5 | 2 |
| 4 | 6 | 4.5 | 1.5 |
| 5 | 11 | 5 | 6 |
| 6 | 12 | 6 | 7 |
| 7 | 6 | 4 | 2 |
| 8 | 6 | 4 | 3 |
| 9 | 25 | 15 | 12 |
| 10 | 29 | 10.5 | 16.5 |
| 11 | 11 | 14 | 11.5 |
| 12 | 17 | 18 | 12 |
| 13 | 10 | 5 | 3.5 |
| 14 | 10 | 5 | 3 |
| 15 | 9 | 5 | 3 |
| 16 | 9 | 5 | 2.5 |
| 17 | 9 | 4 | 4 |


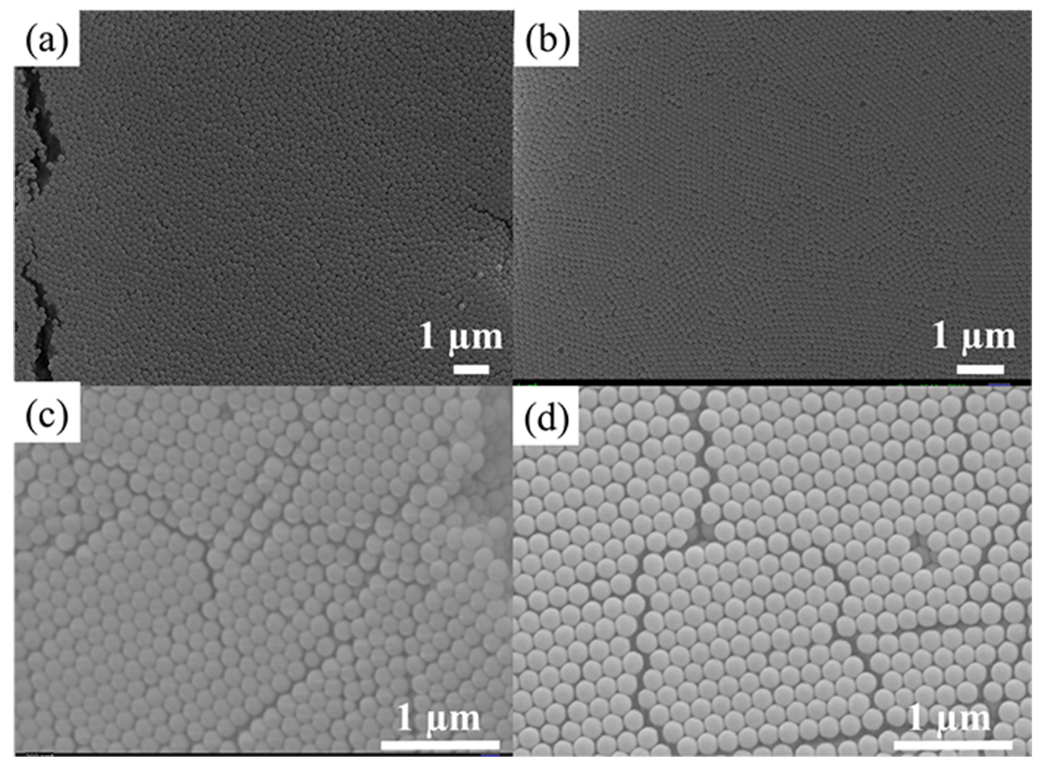


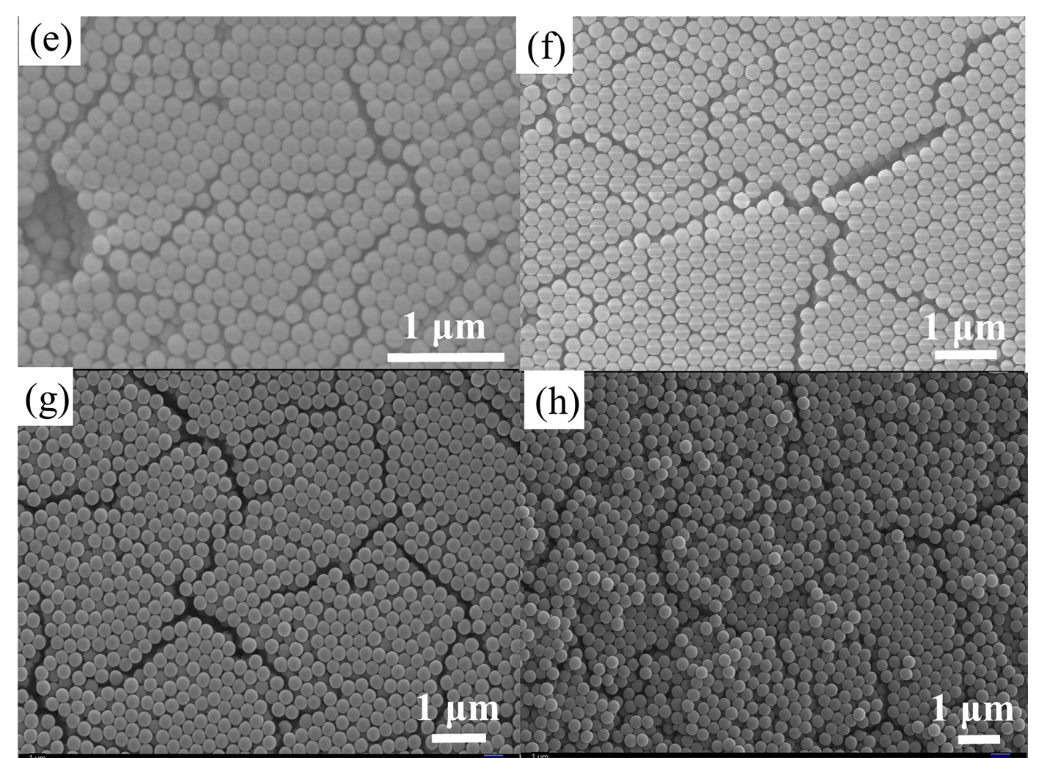


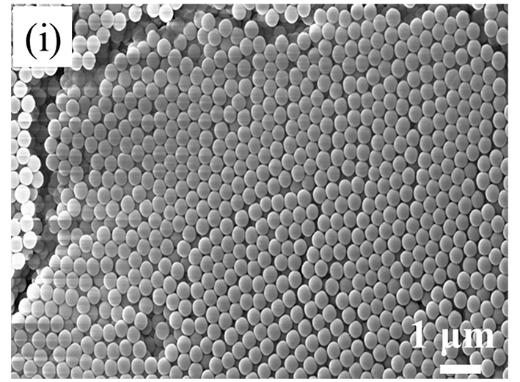


**Figure S1.** SEM images of silica microspheres, (**a**) 105 nm, (**b**) 174 nm, (**c**) 201 nm, (**d**) 201 nm, (**e**) 212 nm, (**f**) 228 nm, (**g**) 283 nm, (**h**) 305 nm and (**i**) 403 nm.

**Table S2.** Statistical analysis of synthesized silica microspheres.

| **Numbers** | **Average Particle Size (nm)** | **Coefficient of Variation** | **Monodispersity** | **Spherical Morphology** | **PDI** |
| --- | --- | --- | --- | --- | --- |
| 1 | 256 | 6.08%±0.67% | good | favorable | 0.019 |
| 2 | 243 | 5.87%±0.37% | good | favorable | 0.027 |
| 3 | 174 | 6.95%±0.48% | good | favorable | 0.033 |
| 4 | 86 | 12.23%±0.76% | bad | unfavorable | 0.201 |
| 5 | 283 | 5.62%±0.84% | good | favorable | 0.018 |
| 6 | 305 | 6.67%±0.86% | good | favorable | 0.016 |
| 7 | 94 | 11.89%±0,68% | bad | unfavorable | 0.143 |
| 8 | 105 | 7.86%±0.42% | good | favorable | 0.037 |
| 9 | 564 | 5.64%±0.21% | good | favorable | 0.018 |
| 10 | 763 | 4.87%±0.27% | good | favorable | 0.023 |
| 11 | 343 | 5.23%±0.32% | good | favorable | 0.016 |
| 12 | 403 | 5.35%±0.38% | good | favorable | 0.029 |
| 13 | 228 | 6.27%±0.28% | good | favorable | 0.017 |
| 14 | 212 | 6.18%±0.52% | good | favorable | 0.030 |
| 15 | 201 | 6.86%±0.33% | good | favorable | 0.021 |
| 16 | 186 | 7.25%±0.41% | good | favorable | 0.013 |
| 17 | 201 | 6.37%±0.24% | good | favorable | 0.020 |


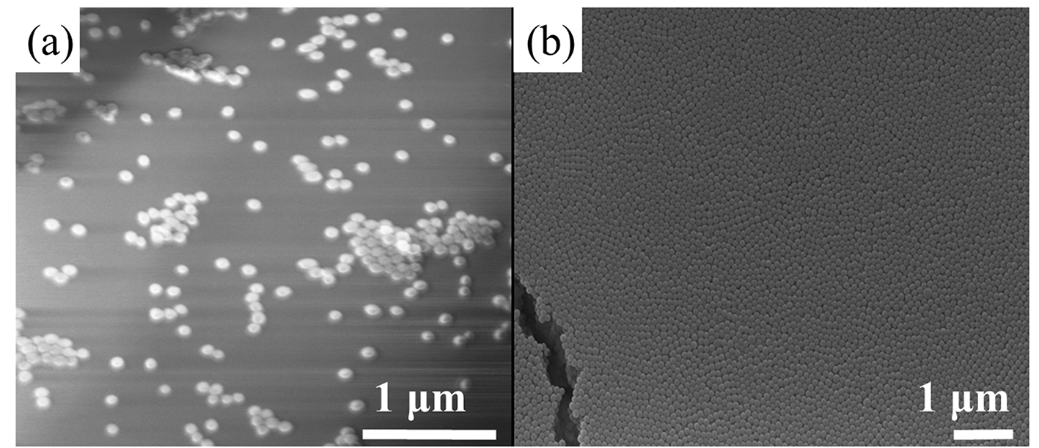


**Figure S2.** SEM images of silica microspheres (a) 66 nm, (b) 78 nm.


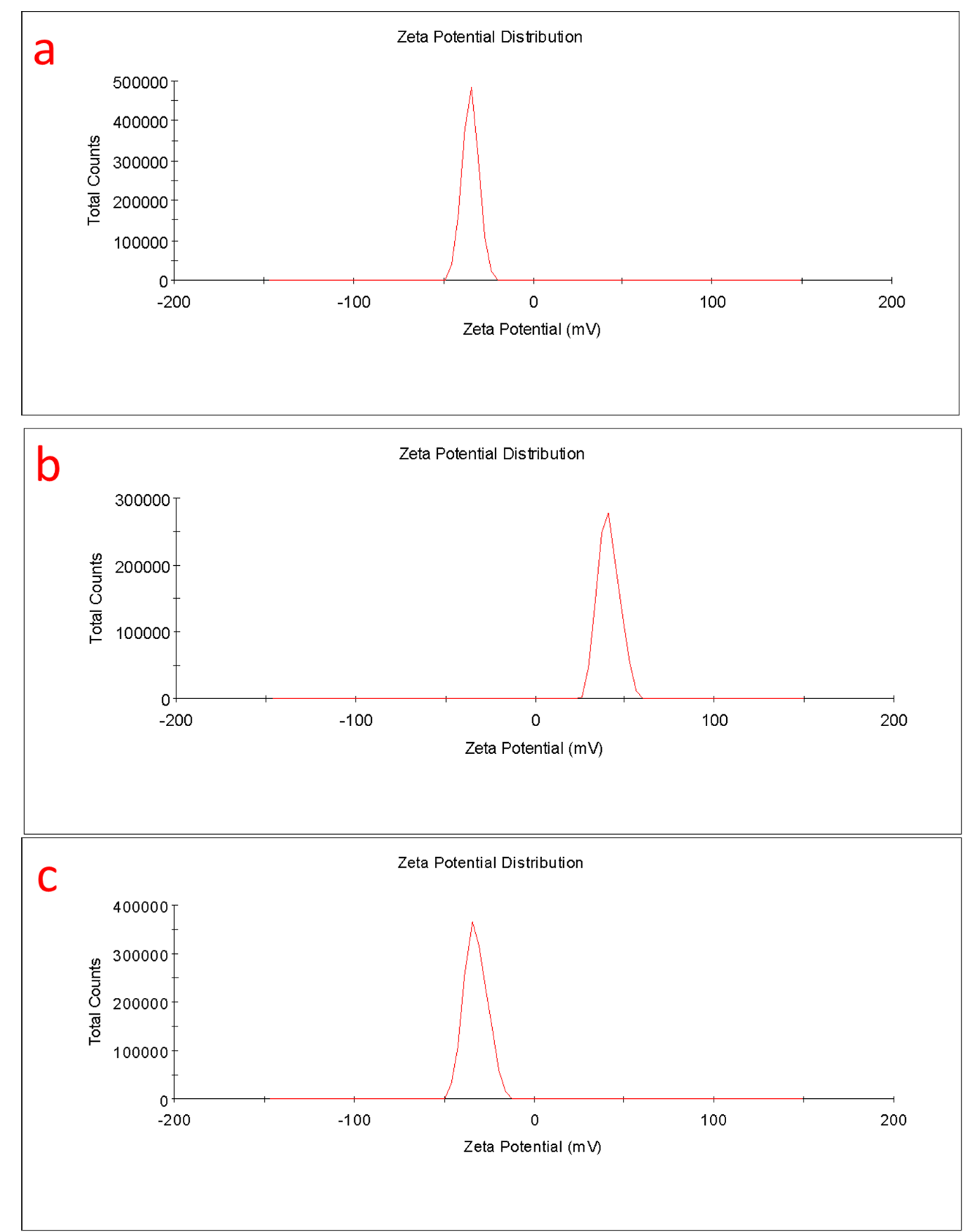


**Figure S3.** Zeta potential changes of silica microspheres before and after surface chemical modification. (**a**) Zeta potential of silica microspheres; (**b**) Zeta potential of aminated silica microspheres; (**c**) Zeta potential of silica microspheres after carbonylation.

**Table S3.** Accuracy analysis of fitting regression equation.

| **Source** | **df** | **Adj SS** | **Adj MS** | **F** | **P** |
| --- | --- | --- | --- | --- | --- |
| Regression | 3 | 471542 | 157181 | 336.75 | 0.000 |
| TEOS (mL) | 1 | 11937 | 11937 | 25.57 | 0.000 |
| Deionized water (mL) | 1 | 3616 | 3616 | 7.75 | 0.01 |
| Ammonia (mL) | 1 | 36940 | 36940 | 79.14 | 0.000 |
| error | 13 | 6068 | 467 |  |  |
| Total | 16 | 477610 |  |  |  |

© 2018 by the authors. Submitted for possible open access publication under the terms and conditions of the Creative Commons Attribution (CC BY) license (http://creativecommons.org/licenses/by/4.0/).
